# A shift from distal to proximal neoplasia in the colon: a decade of polyps and CRC in Italy

**DOI:** 10.1186/1471-230X-10-139

**Published:** 2010-11-25

**Authors:** Luigi Fenoglio, Elisabetta Castagna, Alberto Comino, Cora Luchino, Carlo Senore, Elena Migliore, Franco Capucci, Sergio Panzone, Alberto Silvestri, Luigi Ghezzo, Domenico Ferrigno

**Affiliations:** 1 Medicina Interna, Azienda Ospedaliera S. Croce e Carle, Cuneo, Italy; 2 Anatomia Patologica, Azienda Ospedaliera S. Croce e Carle, Cuneo, Italy; 3 Centro Prevenzione Oncologica, Regione Piemonte e A.S.O. S. Giovanni Battista, Torino; 4 S.S. Endoscopia Digestiva, Azienda Ospedaliera S. Croce e Carle, Cuneo, Italy

## Abstract

**Background:**

In the last years a trend towards proximalization of colorectal carcinomas (CRC) has been reported. This study aims to evaluate the distribution of CRC and adenomatous polyps (ADP) to establish the presence of proximalization and to assess the potential predictors.

**Methods:**

We retrieved histology reports of colonic specimens excised during colonoscopy, considering the exams performed between 1997 and 2006 at Cuneo Hospital, Italy. We compared the proportion of proximal lesions in the period 1997-2001 and in the period 2002-2006.

**Results:**

Neoplastic lesions were detected in 3087 people. Proximal CRC moved from 25.9% (1997-2001) to 30.0% (2002-2006). Adjusting for sex and age, the difference was not significant (OR 1.23; 95% CI: 0,95-1,58). The proximal ADP proportion increased from 19.2% (1997-2001) to 26.0% (2002-2006) (OR: 1.43; 95% CI: 1.17-1.89). The corresponding figures for advanced proximal ADP were 6.6% and 9.5% (OR: 1.48; 95% CI: 1.02-2.17). Adjusting for gender, age, diagnostic period, symptoms and number of polyps the prevalence of proximal advanced ADP was increased among people ≥ 70 years compared to those aged 55-69 years (OR 1.49; 95% CI: 1.032.16). The main predictor of proximal advanced neoplasia was the number of polyps detected per exam (> 1 polyp versus 1 polyp: considering all ADP: OR 2.16; 95% CI: 1.59-2.93; considering advanced ADP OR 1.63; 95% CI: 1.08-2.46). Adjusting for these factors, the difference between the two periods was no longer significant.

**Conclusions:**

CRC do not proximalize while a trend towards a proximal shift in adenomas was observed among people ≥ 70 years.

## Background

Colorectal cancer (CRC) is the third most common cancer and the third leading cause of cancer related mortality in the United States [1]. In Europe, incidence is currently 400,000 new cases/year, with a mortality rate of 200,000/year [2]. CRC is generally a malignancy associated with the elderly, with a mean age at diagnosis of 73 years [3,4]. In the last decade, the literature has reported a change in the topographic distribution of CRC, consisting of a lesion shift towards the proximal sector of the colon [5-7]. Particularly, advanced age [8] and female gender [9] seem to be associated with this phenomenon. Lesion proximalization may have an important impact on clinical practice, as tumours originating from the proximal colon tend to show a better prognosis related both with the high percentage of lesions showing microsatellite instability (MSI) [9], and with the best response to adjuvant chemotherapy [10,11]. Since the majority of CRC arises from adenomatous polyps [12], it is theoretically possible to interrupt this sequence by endoscopic adenomectomy. An increase in the incidence of neoplasms in the right colon might have as well implications for the choice of screening strategies. Indeed, a higher incidence of proximal cancers would tend to reduce the protective effect of sigmoidoscopy (FS), favouring total colonoscopy as the method of choice [13]. We might suppose that the shift of adenomas might precede future proximalisation of CRCs. Our study aims to evaluate the topographic distribution of CRCs and ADPs in our population over a 10 years period in order to assess the occurrence of a trend to the proximalization and to identify those factors associated with the likely proximal shift of these lesions.

## Methods

Our population is represented by a consecutive series of patients, examined at the S. Croce Hospital in Cuneo, Piedmont, with a histological diagnosis of CRC or adenoma between 1997-2001 and 2002-2006. We retrieved histological reports of 2,226 CRCs and 1,858 adenomas. Lesions located between the caecum and the splenic flexure were classified as proximal, while lesions arising in the descending colon, sigmoid and rectum were classified as distal. For patients with synchronous lesions, we considered the most advanced one; in case of metachronos lesions we considered for the analysis the first lesion excised (either adenoma or carcinoma). We did not consider in the analysis hyperplastic polyps, as they were not systematically recorded over the analysed period. Two different databases were designed for carcinomas and adenomas. The carcinoma database included personal data, a macroscopic description [length of resected colon, macroscopic configuration, site, maximum diameter, distance from resection margins, tumour perforation (pT4)], histology [adenocarcinoma or other, differentiation (G), distance between tumour and circumferential margin, neoplastic blood vessel embolization, nerve infiltration], metastases (number of lymph-nodes examined, number of positive lymph-nodes, lymphnodes positivity along the artery, positivity of apical lymph-nodes), background (presence of adenomas or synchronous adenomas), staging (complete margin resection, TNM, staging according to modified Dukes's classification) [14]. Besides personal data, clinical information and symptoms, the adenoma database consisted of variables including colonoscopy examination date, location, morphology (sessile/pedunculate), number (single/multiple), diameter in mm, sample type (biopsy/polypectomy), histological type (tubular/villous/tubulo-villous), grade level of dysplasia (mild/moderate/severe), timing of confirmation of polyp compared with the carcinoma (antecedent/simultaneous/subsequent/during the follow-up period). We identified advanced adenomas (adenomas with a villous component > 20% or a diameter ≥ 10 mm or a high level of dysplasia). It is well known that such lesion is associated with a four fold increase in the risk of neoplasia compared with a low risk lesion (single tubular adenoma < 1 cm) [15]. Since there was no indication concerning the quality of the endoscopic exam (e.g.: the proportion of complete examinations/the different operator/the instrument used), the number of adenomas found per single examination was used as an indicator. For patients with multiple results (those who had undergone more than one colonoscopy in the time period considered) the endoscopic exams performed within 6 months from the first one were considered as complementary to the initial examination and they were evaluated as one exam for the purpose of the analysis. All the colonoscopies were performed without sedation. The quality of bowel preparation was reported in each nursing file: for all the exams the quality of colon cleansing was adequate to permit a complete endoscopic evaluation of the colon. Univariate associations were tested using the χ2 test for proportions. Multivariate logistic regression models were fitted, for CRCs and adenomas separately, to estimate the association (measured as OR) of the prevalence of adenomas and CRCs with the calendar period, adjusting for age, gender, self reported symptoms and number of adenomas detected per each exam (for adenomas only) [16]. All comparisons were considered statistically significant at the 0.05 level (two-sided tests). All analyses were performed using the SAS statistical package.

## Results

**Figure 1:**
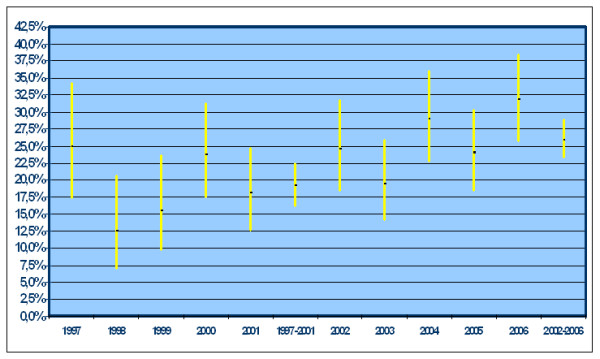
**Prevalence of proximal adenomas per year of diagnosis and in the two periods**.

**Figure 2:**
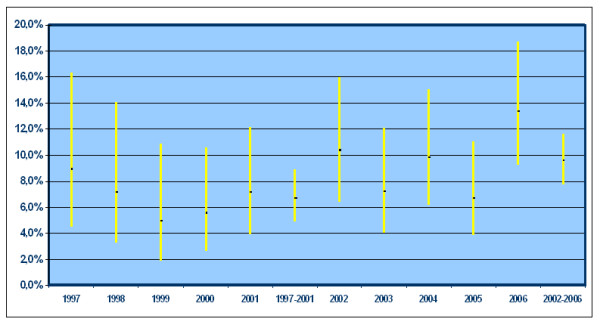
**Prevalence of advanced proximal adenomas per year of diagnosis and in the two periods**.

The actual number of patients was 3,087 (1,361 carcinomas and 1,726 adenomas). We excluded: 659 CRCs and 44 adenomas detected in patients already diagnosed with adenomas in a previous exam. The general characteristics of the population are shown in table 1. ***Carcinomas ***(table 2): among the 1,361 registered cases there were 1,088 surgical resections and 273 colonscopies. The male gender made up 57.8%. Mean age at diagnosis was 68.9 years (range 6277), with a statistically non significant difference between the two genders (68.9 males versus 70.3 females). Excluding 21 reports which did not indicate the tumour site, the proximal carcinomas represented 25.9% of the total number in the first period and 30.0% in the second period respectively, without any statistically significant increase between the two periods (OR 1.23; 95% CI: 0,95-1,58). Adjusting for gender, age and period of diagnosis, the distribution of proximal lesions proved to be significantly higher in the female group; we observed a different trend for age in the two periods, with a greater prevalence of proximal lesions in the younger group in the first period and a tendency to increase in the elderly in the second period. ***Adenomas ***(table 3): among the 1,726 registered cases there were 1,462 polypectomies and 264 biopsies. The median age did not differ between men (65.7 years; range 58-74) and women (66.4 years; range 59-75). The proportion of total proximal adenomas increased from 19.2% in the first period to 26% in the second period (OR 1.43; 95% CI: 1.17-1.89) (Figure 1). Considering only the advanced adenomas, prevalence increased from 6.6% in the first period to 9.5% in the second period (OR: 1.48; 95% CI: 1.02-2.17) (Figure 2). The prevalence of multiple adenomas in the first period proved to be 8.2% while the prevalence in the second period was 23.8%. Adjusting for gender, age, self-reported symptoms, number of adenomas and period of diagnosis, the main predictors of the proximalization of adenomas were the number of ADP detected at each exam (single versus multiple) (OR 2.16; 95% CI: 1.59-2.93) and the diagnosis period (OR 1.33; 95% CI: 1.03-1.70) (table 4). The prevalence of proximal adenomas tended to increase with age, even if the difference did not reach the threshold of statistical significance (OR 1.25; 95% CI: 0.971.60) (table 4). Restricting the analysis to advanced adenomas, given the correlation between the diagnosis period and the number of advanced adenomas found, these two variables were not significant if included together into the logistic model. However, when considering the two variables separately, along with gender, age and symptoms, both the diagnosis period (OR 1.49; 95% CI: 1.03-2.16) and the number of adenomas (OR 1.63; 95% CI: 1.08-2.46) proved to be significantly associated with proximalization (table 5). The prevalence of advanced proximal adenomas increased in subjects ≥ 70 years compared with subjects aged between 55-69 years (OR 1.49; 95% CI: 1.03-2.16).

**Table 1:** Population general characteristics

	1997	1998	1999	2000	2001	2002	2003	2004	2005	2006	Tot
patients	211	220	222	283	289	343	357	377	393	392	3087

mean age ± DS	67.6 ± 8.8	66.6 ± 1	66.5 ± 10.9	67.6 ± 12.7	66.2 ± 4.9	67.6 ± 2.1	67.4 ± 5.6	67.2 ± 6.0	67.0 ± 8.1	66.0 ± 15.5	67.0

M	123	102	95	139	123	154	214	245	248	242	1685

F	88	118	127	144	166	189	143	132	145	150	1402

Total lesions (%)	6.8	7.1	7.2	9.1	9.4	11.1	11.6	12.2	12.7	12.8	100

**Table 2:** Characteristics of patients with carcinoma

	1997	1998	1999	2000	2001	2002	2003	2004	2005	2006	Tot
patients	99	108	100	119	106	161	161	177	168	162	1361

mean age ± DS	70.1 ± 2.12	68.5 ± 0.7	69.1 ± 0.7	68.3 ± 9.9	67.5 ± 3.5	69.4 ± 3.5	69.4 ± 3.5	68.6 ± 1.4	69.2 ± 14.1	69.0 ± 7.1	68.9

M	53	59	55	66	64	84	95	110	103	97	786

F	46	49	45	53	42	77	66	67	65	65	575

carcinomas (%)	7.3	7.9	7.3	8.7	7.8	11.8	11.9	13.0	12.4	11.9	100

**Table 3:** Characteristics of patients with adenoma

	1997	1998	1999	2000	2001	2002	2003	2004	2005	2006	Tot
patients	112	112	122	164	183	182	196	200	225	230	1726

mean age ± DS	65.2 ± 15.5	65.0 ± 1.4	63.9 ± 21.2	67.0 ± 15.5	64.9 ± 6.4	66.4 ± 2.8	65.3 ± 7.8	65.7 ± 10.6	64.9 ± 2.1	63.0 ± 24	65

M	70	43	40	73	59	70	119	135	145	145	899

F	42	69	82	91	124	112	77	65	80	85	827

adenomas (%)	6.5	6.5	7.0	9.5	10.6	10.5	11.3	11.7	13.0	13.4	100

**Table 4:** Multivariate analysis for gender, age, symptoms, total number of adenomas and diagnosis period

	MALES	1
	FEMALES	0.86 (95% CI: 0.69-1.09)

	< 55	0.91 (95% CI: 0.65-1.28)

**AGE**	55-69	1
	> 69	1.25 (95%CI:0.97-1.60)

**SYMPTOMS**	NO	1
	YES	0.67 (95%CI:0.46-0.98)

**DIAGNOSIS PERIOD**	1997-2001	1
	2002-2006	1.33 (95%CI:1.03-1.70)
	1	1
	> 1	2.16 (95%CI:1.59-2.93)

**Table 5:** Determinants of the prevalence of proximal advanced adenomas

	MALES	1	1
	FEMALES	1.21 (95%CI:0.86-1.72)	1.21 (95%CI:0.86-1.72)
	< 55	0.67 (95%CI:0.38-1.19)	0.67 (95%CI:0.38-1.19)

**AGE**	55-69	1	1
	> 69	1.49 (95%CI:1.03-2.16)	1.49 (95%CI:1.03-2.16)

**SYMPTOMS**	NO	1	1
	YES	0.59 (95%CI:0.32-1.10)	0.59 (95%CI:0.32-1.10)
	1997-2001	1	1
	2002-2006	1.36 (95%CI:0.92-2.00)	1.49 (95%CI:1.03-2.16)
	1	1	1
	> 1	1.46 (95%CI:0.95-2.25)	1.63 (95%CI:1.08-2.46)

## Discussion

By age 70 years, at least 50% of the Western population will develop some form of CRC, spanning the spectrum from an early benign polyp to an invasive adenocarcinoma. The stage of disease is one of the most important prognostic factor for CRC patient survival. The incidence of Stage I disease in the USA has increased over the past years due to better screening and is currently nearly 30%. The 5-year survival rates for Stage I colon and rectal cancer are 93% and 92% respectively, while for advanced disease the survival rates drop significantly [17,18]. The most common location of CRC is the sigmarectum (70-75%); about 30% of these lesions is manually explorable, while the other 70% may be diagnosed by sigmoidoscopy. In the last decade, the literature has reported modifications as to the topographic distribution of CRCs [5-7], consisting of the lesions shifting to the proximal sectors of the colon. This proximal shift mainly occurs in industrialized countries, where the incidence of neoplasm is greater. All the current epidemiological studies report a large geographic variation in the anatomic distribution of lesions [19], due to different causes including the impact of environmental risk factors such as diet [20], the difference in the frequency of hereditary CRCs (characterized by a greater prevalence of proximal lesions) [21], and the use of colonoscopy screening [22]. Incidence of proximal neoplasms increases with age [23,24], particularly in the female gender, while distal neoplasms prevail in males. The reason for this seems to lie in the eating habits which may depend on the different hormonal gender set-up [7,21]. In the last years, biomolecular investigations have allowed the identification of different features in proximal and distal lesions, both in pathogenesis and natural history, with different therapeutic strategies [25-30]. Moreover, recent investigations have changed the concept that hyperplastic polyps are innocuous lesions with no potential for progression to malignancy; in fact, we now recognize that the lesions formerly classified as hyperplastic actually represent a heterogeneous group of polyps, some of wich have a significant risk for neoplastic transformation. These serrated polyps include not only hyperplastic polyps but also traditional serrated adenomas and sessile serrated adenomas: these polyps demonstrate characteristic molecular alterations not commonly seen in colorectal adenomas, and they probably progress to colorectal cancer by means of a new pathway: the serrated neoplasia pathway [31]. Many authors have connected carcinoma proximalization with the widespread use of endoscopy, in particular sigmoidoscopy as a screening tool, allowing adenoma removal and a substantial reduction in the incidence of malignant lesions situated in the distal colon and rectum [32,33]. Increase in the incidence of carcinomas in the first segments of the colon thus offers potential implications as regards screening and control of subjects at risk. By increasing the incidence of proximal carcinomas, it seems evident that only FS may not be considered the gold standard as a screening method while total colonoscopy might be the method of choice for CRC screening in subjects at an intermediate risk [34]. To date, in Italy, four epidemiological studies have been performed in order to highlight likely variations in the anatomic distribution of this neoplasm [19,23,35,36] with contrasting results. In a twenty year study (1978-1999) performed in Northern Italy [19], a significant increase both in the incidence of CRCs and proximal lesions was observed, more markedly in the female gender over age 70 years. A second thirty year Italian study (1969-1998) [23] also noted a proximal shift of CRCs. The authors explained this phenomenon as being due to eating habits (greater consumption of fats and protein) and a sedentary lifestyle typical of Western countries [24]. In contrast to the previous two studies, the study published in 2004 [36] and carried out between 1984-1998, showed no significant changes in the CRC distribution, but rather an increase in the incidence of the lesions in all the colon segments, attributed by the authors to the age of the general population. In 2008, a retrospective study [35] was carried out on Turin population between 1992-2001; results showed a reduced prevalence (from 9.9% to 6.8%) with no evidence of proximalization. On the contrary, an increased number of both diagnosed lesions and proximal shifts as regards polyps was found. However, both gender and age did not influence location of carcinomas or polyps, contrary to what had been reported in the literature,. Considering these contrasting results, we wished to retrospectively analyse clinical data of a population resident in the North-West of Italy between 1997-2006. As to carcinomas, there was an increased tendency towards the number of proximal lesions in the two periods, though not statistically significant. It might be presumed that CRC proximalization in our population has not yet occurred. Similar results have been reported in a British study performed in 2004 [7].

Since it is well known that the adenoma-carcinoma sequence takes at least 10 years [37] we hypothesized that an adenoma proximalization is currently occurring in our population. This phenomenon has not yet been pointed out for carcinoma proximalization for the same reason, however, it may occur in the future. Some studies have in fact reported a right sided shift tendency even in adenomatous lesions with increased age [38-40]. Our study showed a significant increase in proximal adenomas between the first (19,2%) and the second five year period (26%). The main predictive factors for proximal location proved to be both the diagnosis period and the number of adenomas detected at each endoscopic exam (single adenoma versus multiple adenoma). On the other hand, age does not seem to be significantly correlated to the proximalization of ADPs of any type even with an increasing trend in the over 70 years age group. Moreover, as to the advanced adenoma subgroup prevalence of proximal location proved to be significantly increased (from 6% to 9.5%) between the first and second period in subjects aged over 70 years. In this setting, the main factors predicting proximalization in the latter case appear to involve the number of lesions detected at each exam (single advanced adenoma vs multiple advanced adenoma). Correlation between the trend in the proportion of advanced proximal adenomas and the proportion of multiple adenomas in the two periods makes difficult the estimation of the independent effect of the diagnosis period. In accordance with the literature, our data also show a proximal shift of precancerous lesions which are partially attributable to patient age and partially to factors associated with diagnostic methods or modifications in the risk profile of the studied population. Increase in the number of cases identified with multiple adenomas is likely due to improved diagnostic accuracy of endoscopic examinations and/or a change in the population referred to this service, with an increase in the amount of people at a higher risk (i.e., familial predisposition). However, we have no further information as to verifying this hypothesis, although the amount of diagnosed lesions is generally considered as an indicator of examination accuracy. Furthermore, as no information concerning the reason for referral nor the total number of examinations performed is available it cannot be ruled out that the variations detected are attributable to changes in the features of the population referred to our centre. The interaction observed between age and diagnosis period of the carcinomas would seem to indicate that the risk profile of the population studied changed over the ten year period considered. The fact that our sample consists of patients referred for various reasons (symptoms such as changes in bowel habits or even familial predisposition) to a hospital unit representing a population at a higher risk than general population for adenomatous polyposis and CRC, sets the main limit of our study. Interest in the study of CRC neoplasia distribution arises from the debate over the choice of screening tests for the intermediate risk population, which is subjects over 50 years without any other risk factors except age. The aim of a screening test consists of reducing the mortality rate by early carcinoma diagnosis and decreasing disease incidence by removal of precursors of invasive disease, mainly advanced adenomas. While the high risk population (familial adenomatous polyposis, Lynch-HNPCC syndrome, inflammatory bowel disease) has a well defined screening test [41], various options are currently available for carrying out a CRC screening test on the intermediate risk population: a) tests carried out on faeces (faecal occult blood test and in the future DNA analysis); b) endoscopic exams (sigmoidoscopy/colonoscopy). Faecal tests fundamentally identify carcinomas although they may result positive in the case of an advanced adenoma, while endoscopic exams can detect carcinomas as well as adenomatous lesions [42].

## Conclusions

In conclusion, in our population there is a non significant trend in the increase of proximal cancers whereas, in accordance with the Western literature, a clear proximal shift of precancerous lesions can be observed affecting the over 70 age group. On the other hand, variations in the proportion of proximal neoplastic lesions observed in the study period would seem at least partially attributable to increased age of the patients examined, as well as to changes in other features of the population referred to our service and/or to improved diagnostic performance. These factors should be taken into consideration in the studies which analyse trend in cancerous and precancerous lesion prevalence in different colon segments.

Due to the characteristics of the study group it is difficult to apply these data to the intermediate asymptomatic risk population, even if this study has not pointed out any trend which could justify debating over current strategies in the target age group of the screening programmes (50-69 years).

## Abbreviations

CRC: colorectal cancer; ADP: adenomatous polyp; OR: odds ratio; CI: confidential interval; FS: flexible sigmoidoscopy.

## Competing interests

The authors declare that they have no competing interests.

## Authors' contributions

LF, EC, CL and CS contributed to study concept and design; EC, CL, EM, AC, FC, AS, LG and SP contributed to acquisition data; CS performed statistical analysis; EC, CL and LF contributed to draft the manuscript; LF, CS, EC, CL and DF analyzed the data; EC, CL and DF wrote the paper; DF performed study supervision.

## Pre-publication history

The pre-publication history for this paper can be accessed here:

http://www.biomedcentral.com/1471-230X/10/139/prepub

## References

[B1] National Cancer Institute A snapshot of Colorectal Cancer 2005 http://www.planning.cancer.gov

[B2] Ferlay J Autier P Boniol M Estimate the cancer incidence and mortality in Europe in 2006 Ann Oncol 2007 18 581 92 10.1093/annonc/mdl498 17287242

[B3] Fahy B Bold RJ Epidemiology and molecular genetics of colorectal cancer Surg Oncol 1998 7 115 123 10.1016/S0960-7404(99)00021-3 10677163

[B4] Parkin DM Bray FI Devesa SS Cancer burden in the year 2000. The global picture Eur J Cancer 2001 37 8 S4 S66 10.1016/S0959-8049(01)00267-2 11602373

[B5] Cady B Stone MD Wayne J Continuing trends in the prevalence of right-sided lesions among colorectal carcinomas Arch Surg 1993 128 505 509 8489383 10.1001/archsurg.1993.01420170035004

[B6] Obrand DI Gordon PH Continued change in the distribution of colorectal carcinoma Br J Surg 1998 85 246 248 10.1046/j.1365-2168.1998.00507.x 9501827

[B7] Gomez D Dalal Z Raw E Anatomical distribution of colorectal cancer over a 10 year a true "rightward shift"? Postgrad Med J 2004 80 667 669 10.1136/pgmj.2004.020198 15537853 PMC1743144

[B8] Okamoto M Stiratori Y Yamaji Y Relationship between age and site of colorectal cancerbased on colonoscopy findings Gastrointest Endosc 2002 55 548 51 10.1067/mge.2002.122335 11923770

[B9] Grady WM Genomic instability and colon cancer Cancer Metastasis Rev 2004 23 11 27 10.1023/A:1025861527711 15000146

[B10] Wils J O'Dwyer P Labianca R Adjuvant treatment of colorectal cancer at the turn of the century: European and US perspectives Ann Oncol 2001 12 13 22 10.1023/A:1008357725209 11249040

[B11] Wils J Adjuvant treatment of colon cancer: past, present and future J Chemother 2007 19 115 122 17434818 10.1179/joc.2007.19.2.115

[B12] Winawer SJ Natural history of colorectal cancer Am J Med 1999 106 3S 6S 10.1016/S0002-9343(98)00338-6 10089106

[B13] Podolsky DK Going the distance - the case for true colorectal cancer screening N Engl J Med 2000 343 207 8 10.1056/NEJM200007203430309 10900282

[B14] Aster VB Coller FA The prognostic significance of direct extension of carcinoma of the *Med *colon and rectum Ann Surg 1957 139 846 10.1097/00000658-195406000-00015 PMC1609522 13159135

[B15] Sobin LH Wittekind CH TNM classification of malignant tumours 2002 Sixth Wiley-Liss, New York

[B16] Cox DR Regression models and life tables J R Stat Soc 1972 34 187 220

[B17] O'Connel JB Maggard MA Ko CY Colon cancer survival rates with the new American JointCommittee on Cancer sixth edition staging J Natl Cancer Inst 2004 96 1420 25 10.1093/jnci/djh275 15467030

[B18] Gloeckler Ries LA Reichman ME Lewis DR Cancer survival and incidence from the Surveillance, Epidemiology, and End Results (SEER) program Oncologist 2003 8 541 52 10.1634/theoncologist.8-6-541 14657533

[B19] Sarli L Dichiara M Sgargi P The changing distribution and survival of colorectal carcinoma: an epidemiological study in an area of northern Italy Eur J Gastroenterol Hepatol 2005 17 567 72 10.1097/00042737-200505000-00014 15827448

[B20] Potter JD Colorectal cancer: molecules and populations J Natl Cancer Inst 1999 91 916 932 10.1093/jnci/91.11.916 10359544

[B21] Lynch HT Smyrk T Lynch J An update of HNPCC (Lynch Syndrome) Cancer Genet Cytogenet 1997 93 84 99 10.1016/S0165-4608(96)00290-7 9062584

[B22] Brenner H Arndt V Sturmer T Long-lasting reduction of risk of colorectal cancer following screening endoscopy Br J Cancer 2001 85 972 76 10.1054/bjoc.2001.2023 11592768 PMC2375093

[B23] Cucino C Parente F Bianchi Porro G Rightward shift of colorectal cancer in Italy during the past three decades Scandinavian J Gastroenterol 2004 39 783 786 10.1080/00365520410005982 15513366

[B24] Okamoto M Stiratori Y Yamaji Y Relationship between age and site of colorectal cancer based on colonoscopy findings Gastrointest Endosc 2002 55 548 51 10.1067/mge.2002.122335 11923770

[B25] Gryfe R Kim H Hsieh ETK Tumor microsatellite instability and clinical outcome in young patients with colorectal cancer N Engl J Med 2000 342 69 77 10.1056/NEJM200001133420201 10631274

[B26] Lukish JP Muro K DeNobile J Prognostic significance of DNA replication errors in youngpatients with colorectal cancer Ann Surg 1998 227 51 6 10.1097/00000658-199801000-00008 9445110 PMC1191172

[B27] Wright CM Dent OF Barker M Prognostic significance of extensive microsatellite instability in sporadic clinicopathological stage C colorectal cancer Br J Surg 2000 87 1197 2002 10.1046/j.1365-2168.2000.01508.x 10971428

[B28] Benatti P Gafa R Barana D Microsatellite instability and colorectal cancer prognosis Clin Cancer Res 2005 11 8332 40 10.1158/1078-0432.CCR-05-1030 16322293

[B29] Carethers JM Smith EJ Behling CA Use of 5-fluorouracil and survival in patients with microsatellite-unstable colorectal cancer Gastroenterology 2004 126 394 401 10.1053/j.gastro.2003.12.023 14762775

[B30] Toyota M Ahuja N Ohe-Toyota M CpG islands methilator phenotype in colorectal cancer Proc Natl Acad Sci USA 1999 96 8681 86 10.1073/pnas.96.15.8681 10411935 PMC17576

[B31] Noffsinger AE Serrated polyps and colorectal cancer: new pathway to malignancy Ann Rev Path 2008 4 343 64 10.1146/annurev.pathol.4.110807.092317 19400693

[B32] Boland CR Savides TJ The changing scope of colorectal cancer Gut 2001 49 449 450 10.1136/gut.48.4.449a PMC1728258 11288733

[B33] Nelson RL Persky V Turyk M Determination of factors responsible for the declining incidence of colorectal cancer Dis Colon Rectum 1999 42 741 52 10.1007/BF02236929 10378598

[B34] Podolsky DK Going the distance - the case for true colorectal cancer screening N Engl J Med 2000 343 207 8 10.1056/NEJM200007203430309 10900282

[B35] Fenoglio L Cena P Cavallo Perin P Proximalisation of colorectal carcinoma: a ten-year study in Italy Dig Dis Sci 2008 53 3 736 40 10.1007/s10620-007-9916-z 17717749

[B36] Ponz de Leon M Marino M Benfatti P Trend incidence, subsite distribution and staging of colorectal neoplasm in the 15-year experience of a specialised cancer registry Ann Oncol 2004 15 940 46 10.1093/annonc/mdh224 15151952

[B37] Morson BC Evolution of cancer of the colon and rectum Cancer 1974 34 845 9 10.1002/1097-0142(197409)34:3+<845::AID-CNCR2820340710>3.0.CO;2-H 4851945

[B38] Yamaji Y Mitsushima T Ikuma H Right-side shift of colorectal adenomas with aging Gastrointest Endos 2006 63 453 58 10.1016/j.gie.2005.09.014 16500395

[B39] Offerhaus GJA Giardiello FM Tersmette AC A shift from distal to proximal neoplasia? Four decades of adenomatous polyps at the Johns Hopkins Hospital Gastroenterology 1990 98 301

[B40] Bernstein MA Feczko PJ Halpert RD Distribution of colonic polyps: Increased incidence of proximal lesions in older patients Radiology 1985 155 35 37 3975415 10.1148/radiology.155.1.3975415

[B41] Levin B Lieberman DA McFarland B Screening and Surveillance for the Early detection of Colorectal Cancer and Adenomatous Polyps CA Cancer J Clin 2008 58 130 160 10.3322/CA.2007.0018 18322143

[B42] Imperiale TF Wagner DR Lin CY Risk of advanced proximal neoplasms in asymptomatic adults according to the distal colorectal findings N Engl J Med 2000 343 169 74 10.1056/NEJM200007203430302 10900275

